# Intraosseous Hemangioma of the Middle Turbinate Misdiagnosed As a Nasal Polyp

**DOI:** 10.1155/2014/217349

**Published:** 2014-08-06

**Authors:** Tae Hoon Kim, Eun Jung Lim, Jun-Ki Lee, Jin Gul Lee, Man-Hoon Han

**Affiliations:** ^1^Department of Otolaryngology-Head and Neck Surgery, Daegu Fatima Hospital, 183 Ayang-ro, Dong-gu, Daegu 701-724, Republic of Korea; ^2^Department of Pathology, Daegu Fatima Hospital, Daegu 701-724, Republic of Korea

## Abstract

Intraosseous hemangiomas account for 1% of all bone tumors and primarily originate from the vertebral column and skull bones. However, intraosseous hemangiomas of the nasal cavity are extremely rare. Here, we report a case of intraosseous hemangioma with a cavernous pattern arising from the middle turbinate that was preoperatively misdiagnosed as chronic rhinosinusitis with polyps. Except for nasal obstruction, there were no specific rhinologic symptoms. The tumor was excised en bloc by the endoscopic endonasal approach without preoperative embolization.

## 1. Introduction

Tumors of the nasal cavity and paranasal sinus have different manifestations including hemangiomas. Hemangiomas are benign vascular tumors and account for about 20% of all benign nasal cavity tumors [1]. Hemangiomas can occur in the skin, mucosa, and deep-seated structures such as bones and muscles. Intraosseous hemangiomas, also known as hemangiomas arising from the primary bone, include about 0.7–1% of all bone tumors [1–3]. Intraosseous hemangiomas primarily originate from the vertebral column and skull bones [1, 3–5]. Several cases of intraosseous hemangiomas of the maxillary sinus and inferior turbinate have been reported in the English literature [1, 4, 6, 7]. However, only 2 cases of intraosseous hemangioma of the middle turbinate have been reported thus far [3, 5]. Here, we report a case of intraosseous hemangioma arising from the middle turbinate that was misdiagnosed as chronic rhinosinusitis with polyps. Similar cases from the literature are also reviewed.

## 2. Case Report

**Figure 1:**
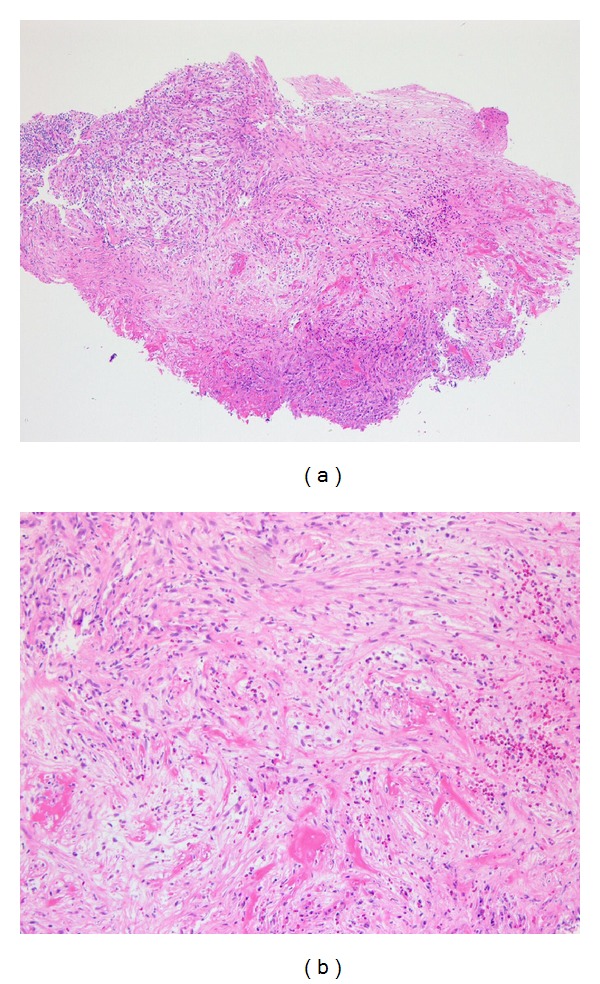
Preoperative histopathologic analysis of the polyp showing fibrinoid necrosis: (a) ×40; (b) ×100.

**Figure 2:**
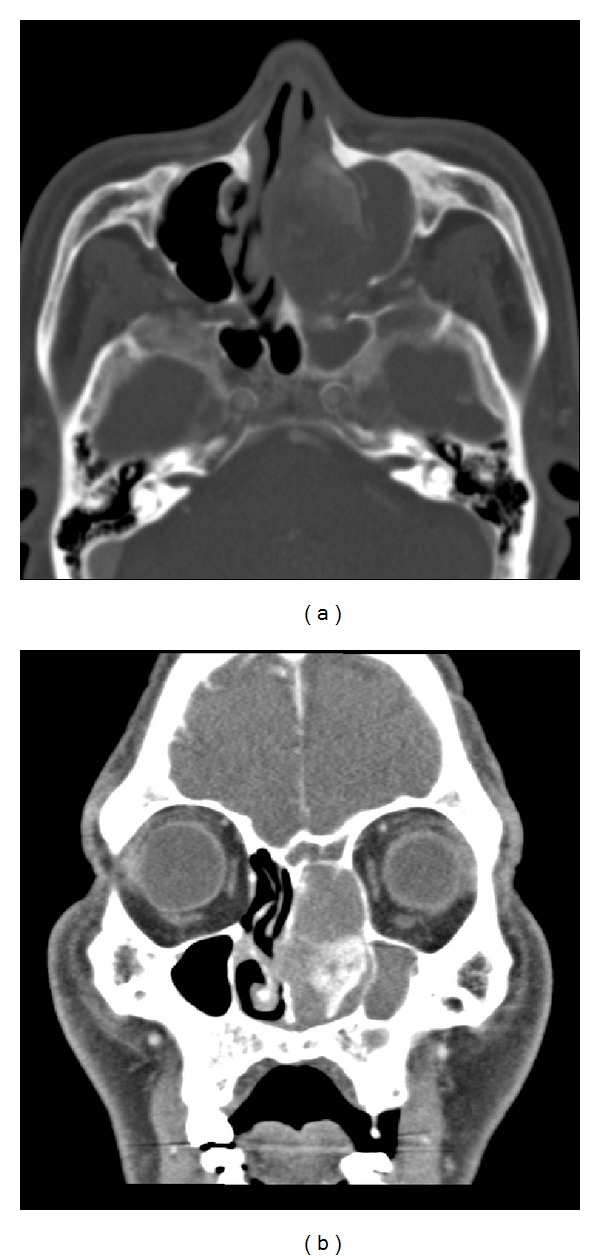
Preoperative computed tomography images showing a mass in the left middle turbinate as well as total opacification in all the sinuses: (a) axial view of the bone window; (b) coronal view of the mediastinum window.

A 64-year-old man with a long history of left nasal obstruction but no epistaxis or facial trauma was referred to our clinic. Anterior rhinoscopy revealed septal deviation to the right and a polyp-like mass occupying the left nasal cavity. We performed an endoscopic biopsy and enhanced computed tomography (CT) at the outpatient clinic. The histologic examination showed the polyp with fibrinoid necrosis (Figure 1). CT scan showed total opacification in all the sinuses as well as lobulated soft tissue mass containing peripheral foci of calcifications. The peripheral intense enhancement after administration of the contrast of media and a lateral displacement of the medial wall of the maxillary sinus due to the mass effect were observed (Figure 2).

**Figure 3:**
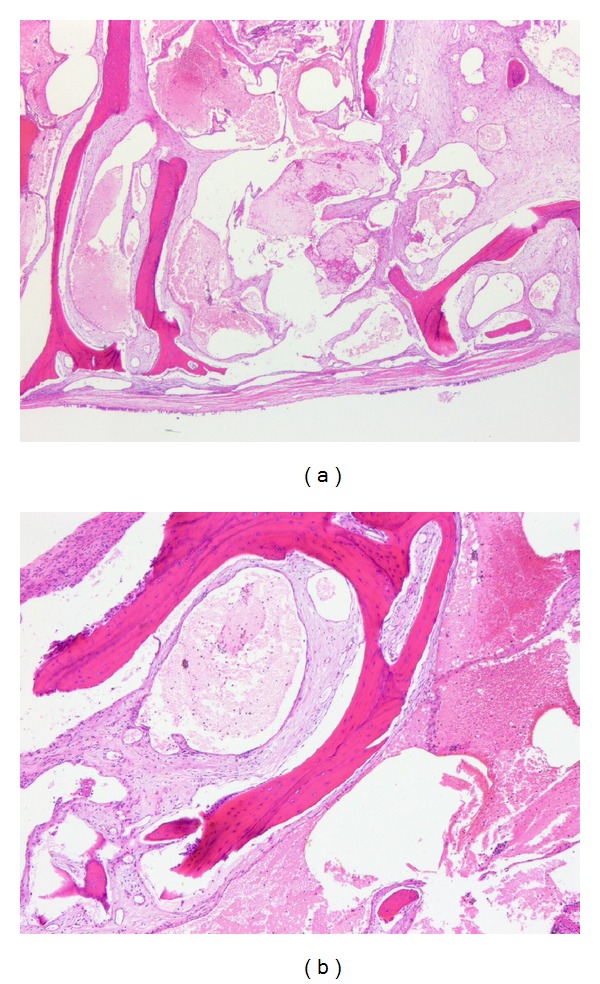
Postoperative histopathologic examinations showing blood-filled, thin-walled vessels between the bony trabeculae: (a) ×20; (b) ×40.

These findings were suggestive of chronic rhinosinusitis combined with either nasal polyps or a benign tumor; therefore, we performed a left endoscopic sinus surgery along with mass excision under general anesthesia. Additionally, a partial middle turbinectomy was performed to remove the mass filling the bottom of the middle turbinate. The tumor was removed transorally after pushing it toward choana. During the surgery, the tumor (5 × 3 × 2 cm) was found to be covered with normal mucosa, with the whole sinus containing mucopurulent discharge and polypoid mucosa. There was no significant bleeding and the left nasal cavity was packed with nonabsorbable materials. The packing was removed on the second postoperative day, after which the patient was discharged home without any complications. The histologic examinations revealed that the tumor comprised anastomosing thin-walled vascular channels with a cavernous pattern between the bony trabeculae and the normal mucosa covering it (Figure 3). Therefore, the patient was diagnosed as having an intraosseous hemangioma with a cavernous pattern. At 24-month follow-up, the patient showed no clinical symptoms, and no recurrence was detected on nasoendoscopic examination.

## 3. Discussion

Hemangiomas are histopathologically classified into 3 types: capillary, cavernous, and mixed. The capillary and cavernous types are divided according to the prominent vessel size. The mixed type hemangiomas comprise a proliferation of thin-walled blood vessels of several sizes lined by endothelium [8].

Hemangiomas of the head and neck are common but those of the nasal cavity and paranasal sinuses are rare. The most common site for nasal hemangiomas is the nasal septum followed by the lateral wall and vestibule [1, 4, 5, 8], and they generally arise from the mucosa. Intraosseous hemangiomas originate from primary bone and usually occur in the vertebrae and skull [1, 3–5], whereas intraosseous hemangiomas of the nasal cavity and paranasal sinuses are rare. Several studies have explained that intraosseous hemangioma is considered vascular anomaly rather than benign tumor [7, 9].

The pathogenesis of intraosseous hemangiomas remains unknown. Nonetheless, local trauma may be a cause, because many patients with intraosseous hemangiomas have had a history of local trauma [1, 3–5]. It has been reported that intraosseous hemangiomas occur predominantly in women in their fourth and fifth decades [1, 3–5]. The most common complaints for nasal hemangiomas are nasal obstruction and epistaxis. The most common symptom for intraosseous hemangiomas is not epistaxis but nasal obstruction due to mass effect [1, 4–6]. Our patient had no history of facial trauma or previous nasal surgery and no specific symptoms except for nasal obstruction, similar to the other cases reported [1, 4–6].

CT is a useful imaging technique for the diagnosis of intraosseous hemangiomas, because of its ability to reveal the characteristic soap bubble or honeycomb patterns [1, 3–6]. Other imaging techniques such as magnetic resonance image (MRI) and angiography are helpful to diagnose and evaluate hemangiomas and are superior to CT in their ability to allow for examination of vascularity. MRI is an effective method for identifying the depth of tumor extension into the soft tissues. Despite being able to identify typical characteristics on CT, the diagnosis of intraosseous hemangiomas is difficult owing to the lack of specific symptoms and the absence of vascular lesions such as the bluish-purple discoloration observed during nasoendoscopic examination. In our case, the patient was misdiagnosed as having chronic rhinosinusitis with nasal polyps because the CT scans showed evidence of pansinusitis as well as no definite characteristic findings such as the soap bubble or honeycomb pattern and the preoperative biopsy demonstrated polyps with fibrinoid necrosis. Therefore, we did not perform any further imaging studies.

The most effective treatment to prevent recurrence of intraosseous hemangiomas is complete surgical resection [1, 3–5]. Other therapeutic options include sclerotherapy and embolization. Sclerotherapy and embolization are used for palliative care. As surgery revealed a solid mass at the level of the middle turbinate, suggesting the presence of a tumor, mass excision including a portion of the middle turbinate was performed. We think that preoperative embolization is not necessary when complete surgical resection could be performed because, on the basis of our experience and several other reports, no significant bleeding occurs during the operation [1, 3–6].

In summary, intraosseous hemangioma of the nasal cavity is an extremely rare disease that is difficult to diagnose without a biopsy. Therefore, it may be necessary to consider a differential diagnosis for intraosseous hemangiomas in the presence of a polyp-like lesion. The typical histological and radiologic characteristics of intraosseous hemangiomas of the nasal cavity will be helpful to make the appropriate diagnosis and effectively manage the condition. Intraosseous hemangiomas arising from the middle turbinate can be excised completely through an endoscopic endonasal approach without the risk of any complications.
